# Editorial: Experience-Dependent Neuroplasticity Across the Lifespan: From Risk to Resilience

**DOI:** 10.3389/fnbeh.2018.00335

**Published:** 2019-01-18

**Authors:** Erica R. Glasper, Gretchen N. Neigh

**Affiliations:** ^1^Department of Psychology, University of Maryland, College Park, MD, United States; ^2^Department of Anatomy and Neurobiology, Virginia Commonwealth University, Richmond, VA, United States

**Keywords:** risk, resilience, neuroplasticity, experience, development

Throughout life, experiences can profoundly shape the structure and function of the brain. This experience-dependent plasticity is observed in numerous cell types, brain regions, and circuits and can contribute significantly to stress regulation, mood, cognition, addiction, etc. This e-book highlights recent efforts in identifying experiences that may confer resilience to or pose a risk for the development of neuropsychiatric disorders and associated alterations in structural plasticity within the brain.

The importance of risk and resilience to long-term sequela of perturbations has been previously reviewed with regard to synaptic plasticity (Hyer et al., 2018), immune function (Bekhbat and Neigh, 2018a,b), and stress responsivity (Bourke et al., 2012; Bekhbat et al., 2017). The mechanisms which underlie the manifestation of risk and resilience following stressor exposure are not fully defined, but much progress has been made in individual disease states and conditions (Nemeth et al., 2014, 2015; Hodes et al., 2016; Neigh and Ali, 2016; Valdez et al., 2016). The mini-review in this e-book by Mukhara et al. focuses on the progress that has been made in terms of identification of candidate molecular mediators in the context of addiction and sets the framework for potential mechanistic studies. In addition to the importance of glucocorticoids and the dopaminergic system in the generation of risk and resilience, inflammatory-sensitive mechanisms within the brain have been identified as a key area of importance in the study of risk and resilience. The review by Finnell and Wood examines risk and resilience in the context of depression, and the authors further highlight the role of individual differences, first introduced in this e-book by Murthy and Gould, in the potential manifestation of risk vs. resilience with a focus on age, sex, and coping strategies. Further, the authors review mechanisms by which inflammatory cytokines and chemokines can alter function of neurons and glial cells precipitating changes in behavior.

Importantly, mechanistic drivers and the relative factors that produce risk and resilience are sensitive to developmental timing and level of exposure. The original research reports highlighted in this e-book span the developmental timeline and guide future inquiry into viable means by which to mitigate risk and produce resilience.

Experiences with offspring greatly influence the parental brain (Leuner et al., 2010), while parental care also influences offspring developmental (Rilling and Young, 2014; Bales and Saltzman, 2016). Traditional parenting-related plasticity is studied in the context of the dam and how interactions with offspring shape their risk or resilience throughout life. Using the biparental California mouse (*Peromyscus californicus*), Yohn et al. demonstrate that neuropeptide levels in social areas of the brain, and gonadal steroid hormones in females, are influenced by the amount of care provided by the father. These data contribute to a growing body of literature that suggests social behaviors, like paternal care, can program the developing brain via lasting effects on the neuroendocrine system.

Conversely, when paternal care is necessary for offspring survival and typical development, the lack of paternal care may increase susceptibility to mood disorders-similar to maternal models of early-life stress (Chen and Baram, 2016). Using paternal deprivation as a model of early-life adversity, Glasper et al. examined hippocampal plasticity of California mice during adulthood. Lack of paternal care increased anxiety-like behavior and behavioral despair in male and female offspring, however, cell survival of adult born cells in the dentate gyrus of the hippocampus was only reduced in paternally-deprived females. This apparent sex-difference in hippocampal structural plasticity following paternal deprivation contributes to our understanding of early-life stress reprogramming of neural regions involved in emotion (Chen and Baram, 2016) in a novel way, and along with Yohn et al. adds to the growing literature on sex differences in neural responsiveness to paternal care.

Adolescence is a time of great change in terms of the structure and function of the nervous system, including decreased cell proliferation and adult neurogenesis in the dentate gyrus of the hippocampus. Adolescence may increase susceptibility to stress-related perturbations during this time of vast neurobiological change. Shome et al. demonstrate that chronic treatment with exogenous glucocorticoids confers sex-specific effects on dentate gyrus structural plasticity. Specifically, chronic corticosterone treatment does not alter cell genesis or cell survival in females, and the effects in males are limited to immature neurons. This work suggests that adolescence-induced reductions in cell genesis may increase resiliency at a time of great environmental perturbations in a sex-specific way.

Medical experiences and somatic illnesses can further contribute to the manifestation of individual differences in susceptibility to stress-related behaviors. Caulfield et al. demonstrate the power of developmental asthma to alter both lung function and induce profound and enduring changes in the stress response system including altered gene expression in the brain and changes in stress-related behaviors. Further, this work demonstrates that individual differences prior to the manifestation of developmental asthma influence the long-term effects of developmental asthma and empirically highlight the important role of individual differences introduced by Murthy and Gould and Finnell and Wood.

Finally, environmental exposures are an important source of individual variability that can drive the manifestation of later-life somatic and mental health conditions. To this end, Pistoia et al. demonstrate the powerful influence of being exposed to the traumatic stress of a substantial earthquake during the adolescent period. Individuals from earthquake-affected areas exhibited an increase in anxiety and increased anticipation of threats including a more vigilant awareness of facial expressions. This inherent pattern of individual difference created in those from earthquake-affected areas could drive a susceptibility to future insults.

Collectively, the work presented in this e-book demonstrates that the manifestation of and mechanisms by which individuals respond to environmental stimuli, ranging from somatic conditions to environmental stressors, is shaped by experiences across the lifespan. These experience-induced changes shape the neural and somatic response to new challenges and exposures. This collection demonstrates that it is essential to consider the collective experiences and exposures of an organism when trying to predict risk vs. resilience. Furthermore, the salient effects of environmental exposures on lasting neural and somatic substrates should be considered when working to treat somatic and neuropsychiatric disorders.

## Author Contributions

EG and GN contributed to the conceptualization of the research topic and to the writing of this editorial.

### Conflict of Interest Statement

The authors declare that the research was conducted in the absence of any commercial or financial relationships that could be construed as a potential conflict of interest.
